# Transferable neural wavefunctions for solids

**DOI:** 10.1038/s43588-025-00872-z

**Published:** 2025-10-22

**Authors:** L. Gerard, M. Scherbela, H. Sutterud, W. M. C. Foulkes, P. Grohs

**Affiliations:** 1 Faculty of Mathematics, University of Vienna, Vienna, Austria; 2 Department of Physics, Imperial College London, London, UK; 3 Johann Radon Institute for Computational and Applied Mathematics, Austrian Academy of Sciences, Linz, Austria

**Keywords:** Computational science, Chemical physics

## Abstract

Deep-learning-based variational Monte Carlo has emerged as a highly accurate method for solving the many-electron Schrödinger equation. Despite favorable scaling with the number of electrons, ${\mathcal{O}}({{n}_{{\rm{el}}}}^{4})$, the practical value of deep-learning-based variational Monte Carlo is limited by the high cost of optimizing the neural network weights for every system studied. Recent research has proposed optimizing a single neural network across multiple systems, reducing the cost per system. Here we extend this approach to solids, which require numerous calculations across different geometries, boundary conditions and supercell sizes. We demonstrate that optimization of a single ansatz across these variations significantly reduces optimization steps. Furthermore, we successfully transfer a network trained on 2 × 2 × 2 supercells of LiH, to 3 × 3 × 3 supercells, reducing the number of optimization steps required to simulate the large system by a factor of 50 compared with previous work.

## Abstract (web summary)

Investigating crystalline materials often requires calculations for many variations of a system, substantially increasing the computational burden. By training a transferable neural wavefunction across these variations, the cost can be reduced by approximately 50-fold for systems such as graphene and lithium hydride.

## Main

Many interesting material properties, such as magnetism and superconductivity, depend on the material’s electronic structure as given by the ground-state wavefunction. The wavefunction may in principle be found by solving the time-independent Schrödinger equation, but doing so with sufficient accuracy is challenging because the computational cost grows dramatically with the number of particles. The challenge is particularly pronounced in solid state physics, where accurate calculations for periodic systems require the use of large supercells—and, consequently, many particles—to minimize finite-size effects.

Over the past few decades, density functional theory (DFT) has emerged as the primary workhorse of solid-state physics. When using local or semi-local exchange–correlation functionals, DFT calculations have a favorable scaling of ${\mathcal{O}}({{n}_{{\rm{el}}}}^{3})$ or better, where *n*_el_ is the number of electrons in the system, and an accuracy that is often sufficient to help guide and predict experiments^1,2^. However, the choice of functional is in practice an uncontrolled approximation, and DFT sometimes yields quantitatively or even qualitatively wrong results, especially for strongly correlated materials^3,4^.

**Fig. 1:**
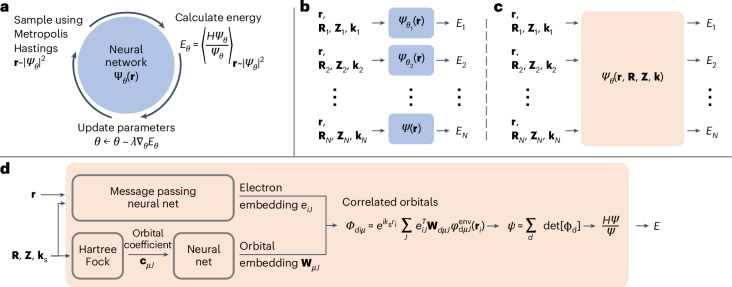
Schematic overview of our approach. **a**, A schematic overview of the VMC optimization loop. **b**, The conventional approach of training separate, geometry- and twist-specific wavefunctions. **c**, Our approach of training a single, transferable wavefunction across system variations. **d**, A schematic of our transferable wavefunction ansatz: starting from electron and nucleus coordinates, nucleus charges **r**, **R**, **Z** and twist **k**_s_, we compute high-dimensional representations **e**_*i*_ for each electron *i* and **W**_*μ*_ for each orbital *μ*. We combine them to square matrices *Φ*_*d*_ and use determinants to obtain an antisymmetric wavefunction *Ψ*_*θ*_ with trainable parameters *θ*. From that, we compute the total energy *E*, applying the Hamiltonian operator *H* to the wavefunction.

Another approach, known as variational Monte Carlo (VMC), uses an explicit parameterized representation of the full many-body wavefunction and optimizes the parameters using the variational principle. This method has a favorable scaling of ${\mathcal{O}}({{n}_{{\rm{el}}}}^{3-4})$ (refs. ^5,6^) but is limited in accuracy by the expressivity of the ansatz used. Recently, deep neural networks have been used as wavefunction ansatze^6–8^ and used to study a large variety of systems including small molecules^6,9,10^, periodic model systems described by lattice Hamiltonians^7,11–13^, the homogeneous electron gas^14,15^ and Fermi liquids^16,17^. Due to their flexibility and expressive power, deep-learning-based VMC (DL-VMC) approaches provide the best current estimates for the ground-state energies of several small molecules^9,10^ In DL-VMC, the wavefunction ansatz *ψ*_*θ*_ is represented as a neural network, with the variational parameters *θ* being the network weights and biases. An approximation of the ground state is obtained by minimizing the energy expectation value of this ansatz (Fig. 1a). In each optimization step, electron coordinates **r** are sampled from the probability density ∣*ψ*_*θ*_∣^2^, and these samples are used to estimate the energy expectation value *E*_*θ*_. Using automatic differentiation, the energy gradient is computed, and the network parameters *θ* are updated to minimize this energy.

Despite the success for small molecules, efforts to apply DL-VMC to real solids^18,19^ have been limited by the high computational cost involved. While a single calculation may be feasible, studying real solids requires many similar but distinct calculations. First, it is necessary to perform calculations involving increasingly larger supercells to estimate finite-size errors and extrapolate results to the thermodynamic limit (TDL). Second, twist-averaged boundary conditions (TABC) are used to accelerate the rate at which the finite-size errors reduce as the supercell size increases^20^. This requires averaging the results for each supercell over many calculations using different boundary conditions. Lastly, studying a given system often requires calculations for different geometries and lattice constants. As most existing DL-VMC ansatze require optimizing a new wavefunction from scratch for each new system (Fig. 1b), the computational cost quickly becomes prohibitive even for systems of moderate size. For example, Li et al. proposed DeepSolid^18^, an ansatz capable of accurately modeling periodic wavefunctions with up to 100 electrons, but it required over 80,000 GPU hours to study a single system.

In this work, we implement a transferable DL-VMC ansatz for real solids that takes as input not only the electron positions but also other parameters of the system, such as its geometry or boundary condition. When computing energies for multiple systems, we do not optimize separate ansatze for each system, but instead optimize a single wavefunction able to represent all these systems (Fig. 1c). The transferability of this wavefunction across systems yields two large speed-ups in practice. First, optimizing a single ansatz for many variations of unit-cell geometry, boundary condition and supercell requires typically much fewer optimization steps than optimizing ansatze separately for each system. Second, because the ansatz learns to generalize across systems, we can use models pretrained on small systems as highly effective initializers for new systems or larger supercells. The key idea, based on Scherbela et al.^21^, sketched in Fig. 1d and detailed in the Methods, is to map computationally cheap, uncorrelated mean-field orbitals to expressive neural network orbitals that depend on the positions of all electrons.

Compared with previous DL-VMC work without transferability, our approach yields more accurate results, gives access to denser twist averaging (reducing finite-size effects) and requires a fraction of the computational resources. For example, for lithium hydride, transferring a 32-electron calculation to one with 108 electrons yields more accurate results than previous work^18^ at approximately 1/50 of the computational cost.

## Results

### One-dimensional hydrogen chains

Chains of hydrogen atoms with periodic boundary conditions provide a simple one-dimensional toy system that nevertheless exhibits rich physics such as dimerization, a lattice-constant-dependent metal–insulator transition and strong correlation effects. A collaborative effort^3,22^ has obtained results for this system using a large variety of high-accuracy methods, providing a trustworthy benchmark.

The first test is to obtain the total energy per atom for a fixed atom spacing, *R* = 1.8*a*_0_ (where *a*_0_ is the Bohr radius), in the TDL attained as the number of atoms in the supercell tends to infinity. To this end, we train two distinct models on periodic supercells with *N*_atoms_ = 4, 6, …, 22. The first model is trained at twist *k* = 0 (the *Γ*-point) only. The second is trained using all twists from a *Γ*-centered four-point Monkhorst–Pack grid^23^. The three inequivalent twists are $k=0,\frac{1}{4}\,\rm{and}\,\frac{1}{2}$ in units of 2π/*R*, and their weights are *w* = 1, 2 and 1, respectively. Once the model has been pretrained on these relatively short chains, we fine-tune it on larger chains with *N*_atoms_ = 32 and 38. We use the extrapolation method described in ref. ^22^ to obtain the energy *E*_*∞*_ in the TDL. Previous authors have extrapolated the energy using only chain lengths of the form ${N}_{{\rm{atoms}}}=4n+2,\ n\in {\mathbb{N}}$, which have filled electronic shells. We also report extrapolations using chain lengths *N*_atoms_ = 4*n*, which lead to partially filled shells.

**Fig. 2:**
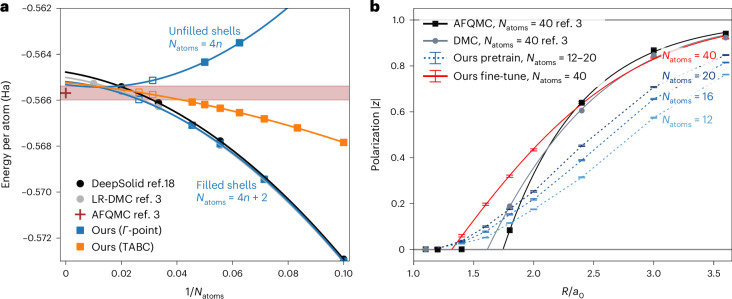
One-dimensional hydrogen chain. **a**, Extrapolation of the energy per atom to the TDL for *R* = 1.8*a*_0_. Results obtained using DeepSolid (neural wavefunction), LR-DMC, AFQMC and our transferable neural wavefunction are shown. Open markers indicate energies computed by fine-tuning a model pretrained on smaller supercells. The shaded area depicts the statistical uncertainty in the AFQMC result. The Monte Carlo uncertainty of our results is approximately 10 μHa, well below the marker size. **b**, The complex polarization ∣*z*∣ as a function of the interatomic separation, *R*, showing a phase transition between a metal at small *R* and an insulator at large *R*. AFQMC and DMC results are taken from the work of the Simons Collaboration^3^. The error bars for our results represent Monte Carlo uncertainty. DeepSolid results are taken from Li et al.^18^. Source data

Figure 2a shows that all of our extrapolations (*Γ*-point filled shells, *Γ*-point unfilled shells and TABC) are in good qualitative agreement with previous results obtained using methods such as lattice-regularized diffusion Monte Carlo (LR-DMC)^22^ and DeepSolid^18^, a FermiNet-based^6^ neural wavefunction for solids. Quantitatively, we achieve slightly lower (and, thus, more accurate) energies than DeepSolid for all values of *N*_atoms_. Using TABC, we obtain *E*_*∞*_ = −565.24(2) mHa, which is 0.2–0.5 mHa lower than the estimate obtained using LR-DMC and DeepSolid, and agrees within uncertainty with the extrapolated energy computed using the auxiliary-field quantum Monte Carlo (AFQMC) method^22^. Most notably, however, we obtain these results at a fraction of the computational cost of DeepSolid. Whereas DeepSolid required a separate calculation with 100,000 optimization steps for each value of *N*_atoms_ (and would have required even more calculations for twist-averaged energies), we obtain results for all 10 chain lengths and values of *N*_atoms_ = 4, …, 22, with 3 twists for each system, using only 50,000 optimization steps in total. Furthermore, by reusing the model pretrained on smaller chains, we obtain results for the larger chains with *N*_atoms_ = 32 and 38 using only 2,000 additional steps of fine tuning. This reduces the cost of simulating the large chains by a factor of approximately 50. We note that, as expected, the use of TABC reduces finite-size errors, allows us to combine results for filled and unfilled shells in the extrapolation and leads to faster convergence of the energy per atom. By contrast, when using only *Γ*-point calculations, there is a strong even/odd effect in the energy, requiring separate extrapolations for unfilled and filled shells.

Beyond energies, we study the hydrogen chain’s phase transition from an insulating phase at large interatomic separation, *R*, to a metallic phase at small *R*. The transition can be quantified by evaluating the complex polarization along the length of the chain

1 $$z=\left\langle {{\mathrm{e}}}^{i\frac{2\uppi }{R{N}_{{\rm{atoms}}}}\mathop{\sum }\limits_{i = 1}^{{n}_{{\rm{el}}}}{x}_{i}}\right\rangle ,$$

where *x*_*i*_ is the position of electron *i* in the direction of the chain. The expectation value is defined as 〈…〉 ≡ ∫Ψ^*^(**r**)…*Ψ*(**r**)d**r**, where ${\boldsymbol{r}}=({{\boldsymbol{r}}}_{1},{{\boldsymbol{r}}}_{2},\ldots ,{{\boldsymbol{r}}}_{{n}_{{\rm{el}}}})$ is a 3*n*_el_-dimensional vector of electron positions, *Ψ* is the (approximate) ground-state wavefunction, and the integral is over all 3*n*_el_ electronic degrees of freedom. Although the polarization is easy to evaluate in principle, studying the transition is computationally costly because it requires many similar but distinct calculations: multiple values of *R* are required to locate the transition; multiple twists *k* are required to obtain accurate twist-averaged polarizations; and multiple chain lengths *N*_atoms_ are required to allow extrapolation to the TDL. Even for a modest selection of all of these variations, studying the phase transition in detail requires hundreds of calculations. Using our transferable wavefunction, on the other hand, allows us to train a single model to represent the wavefunction for all parameter variations at once.

We trained a single ansatz to describe all 120 combinations of: (1) 3 distinct chain lengths, *N*_atoms_ = 12, 16 and 20; (2) 5 symmetry-reduced *k*-points of an 8-point *Γ*-centered Monkhorst–Pack grid; and (3) 8 distinct atom spacings between *R* = 1.2*a*_0_ and *R* = 3.6*a*_0_. A total of 200,000 optimization steps were carried out, after which the complex polarization was evaluated using equation (1). To improve our estimates for *N*_atoms_ → *∞*, we fine-tuned this pretrained model for 2,000 steps on chain lengths of *N*_atoms_ = 40 and a denser 20-point Monkhorst–Pack grid containing 11 symmetry-reduced twists. Figure 2b shows that our approach qualitatively reproduces the results obtained using DMC and AFQMC. In agreement with Motta et al.^3^, we observe a second-order metal–insulator transition. However, where Motta estimates the critical atom spacing *R*_crit_ = 1.70(5)*a*_0_, our results are more consistent with *R*_crit_ = 1.32(5)*a*_0_. A possible explanation for the disagreement is that our neural wavefunction may be less accurate (and may therefore produce relatively higher energies) for metals than insulators, disfavoring the metallic phase. Another possible explanation follows from the observation that, unlike the VMC method used here, the DMC and AFQMC methods yield biased estimates of the expectation values of operators, such as the complex polarization, that do not commute with the Hamiltonian^5,24^.

Also in agreement with Motta et al.^3^, we find that the hydrogen chain shows quasi-long-range antiferromagnetic correlation at at large lattice constant *R*. The expected atomic spins are zero on every atom, but the spins on neighboring atoms are antiferromagnetically correlated. As the lattice constant gets smaller and the system transitions to the metallic phase, these correlations decrease as shown in Supplementary Fig. 3.

### Graphene

**Fig. 3:**
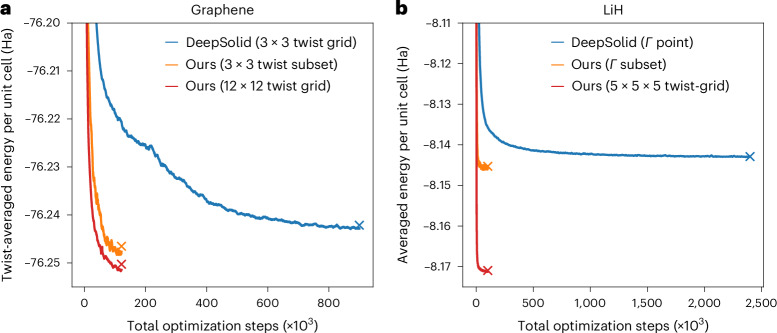
Optimization curves. Mean energy as a function of total optimization steps across all geometries and twists. Energies are the running average over the last 1,000 steps. Crosses mark final evaluation energies. **a**, Energy of 2 × 2 supercell of graphene. **b**, Mean energy of the potential energy surface of LiH in a 2 × 2 × 2 supercell. Source data

To demonstrate the application of our transferable DL-VMC ansatz to a two-dimensional solid, we compute the cohesive energy of graphene in a 2 × 2 supercell and compare against the DL-VMC results of DeepSolid by Li et al.^18^. We use TABC, apply structure-factor-based finite-size corrections^25^ as detailed in the Methods and add zero-point vibrational energies (ZPVE). The DeepSolid results were restricted to a Monkhorst–Pack grid of 3 × 3 twists, yielding three symmetry-reduced twists in total. In our case, because we are able to compute multiple twists at once with minimal extra cost, we increase the grid density to 12 × 12. This increases the number of symmetry-reduced twists from 3 to 19. Our denser twist grid contains a subset of the twists considered by DeepSolid, allowing a direct comparison with their independent energy calculations. We stress that we require only a single neural network, optimized for 120,000 steps, to obtain energies for all twists (both the 12 × 12 grid and the 3 × 3 subset). DeepSolid, on the other hand, optimized for 900,000 steps in total, obtaining energies only for the 3 × 3 twist grid. We find that our transferable ansatz has an approximately 2× higher per-step cost compared with DeepSolid (Supplementary Fig. 2), but the large reduction in the required number of optimization steps (Fig. 3a) far outweighs this cost.

Our twist-averaged energy using the 3 × 3 twist grid is 4 mHa per primitive cell lower than the DeepSolid energy. Looking at individual twists (Table 1), we find that our energies for *k*_1_ and *k*_2_ are lower than the energies obtained by DeepSolid by 1 mHa and 7 mHa, respectively, while our energy for *k*_3_ is higher by 4 mHa. This twist-dependent accuracy is expected, because we allocate optimization steps proportional to the twist’s symmetry weight (see ‘Sampling’ in the Methods), thereby potentially optimizing *k*_1_ and *k*_2_ more stringently than *k*_3_. This procedure ensures that more optimization steps are spent on twists with high contribution to the final energy, thus improving efficiency.

**Table 1:** Total energies of graphene in Hartrees for a primitive cell, as computed by VMC, after the structure factor correction (SFC) and after adding ZPVE

	Twist	Weight	Total energy	Total energy + SFC	Total energy + SFC + ZPVE
DeepSolid	*k*_1_ = (0, 0)	1/9	−76.1559	−76.1534	−76.1406
	*k*_2_ = (1/3, 1/3)	2/3	−76.2495	−76.2470	−76.2342
	*k*_3_ = (2/3, 1/3)	2/9	−76.2631	−76.2607	−76.2479
Our work	*k*_1_ = (0, 0)	1/9	−76.1572(2)	−76.1542(2)	−76.1414(2)
	*k*_2_ = (1/3, 1/3)	2/3	−76.2572(2)	−76.2543(2)	−76.2415(2)
	*k*_3_ = (2/3, 1/3)	2/9	−76.2590(2)	−76.2560(2)	−76.2432(2)

The table compares our results against the total energies computed with DeepSolid^18^ at the three symmetry-inequivalent twists on the 3 × 3 Monkhorst–Pack grid. The twists are expressed in the basis of the reciprocal lattice vectors.

To check for finite size effects, we also compute cohesive energies on a larger 3 × 3 supercell with a 12 × 12 twist grid. Due to the transferability of our wavefunction, we can use wavefunction parameters obtained from the 2 × 2 supercell as initialization for the 3 × 3 supercell calculation, thereby reducing the number of required optimization steps.

When computing cohesive energies and correcting for finite-size effects using a structure-factor-based correction and ZPVE, we obtain energies that are 7 mHa lower than experimental values for the 2 × 2 supercell, that is, we predict slightly stronger binding than experiment. For the 3 × 3 supercell, we predict 15 mHa higher energies than experimental values (Supplementary Table 1). We hypothesize that the remaining discrepancy may be a finite-size artifact and that even for the 3 × 3 supercell energies may not yet be converged. An alternative hypothesis is that a larger, more expressive network may be needed to represent the true ground-state wavefunction for the 3 × 3 supercell.

**Fig. 4:**
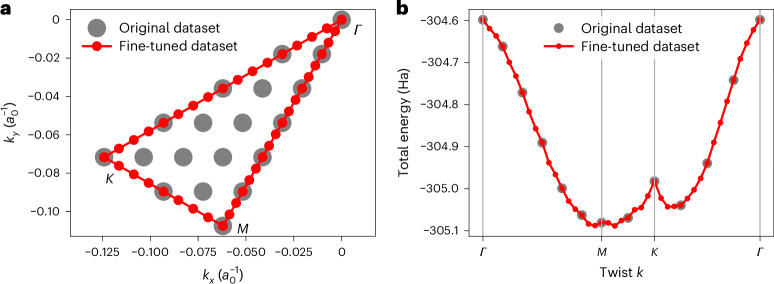
Twist-dependent energy of Graphene. **a**, Grid of pretrained twists and path of fine-tuned values through Brillouin zone. **b**, Fine-tuned energies of graphene along the path of twists across the Brillouin zone, computed using shared optimization and around 100 additional optimization iterations per twist. The error bars are smaller than the size of the markers. Source data

With a network that has been trained across the entire Brillouin zone, we can evaluate observables along arbitrary paths in *k* space. Figure 4 is a bandstructure-like diagram, showing how the total energy varies along a path passing through the high-symmetry *k*-points *Γ* = (0, 0), *M* = (0, 1/2) and *K* = (1/3, 2/3) in units of the supercell reciprocal lattice vectors. We use the pretrained model from the 12 × 12 Monkhorst–Pack grid and transfer it to the bandstructure-like diagram with *k*-points previously unseen during optimization, requiring only a few additional optimization steps. We fine-tune the pretrained model for the *k*-points on the path, using around 100 optimization steps per twist and then evaluate the energies along the path. Analogously to the Dirac cone visible in the one-electron bandstructure, our many-electron bandstructure displays a characteristic cusp at the *K*-point.

### Lithium hydride

**Fig. 5:**
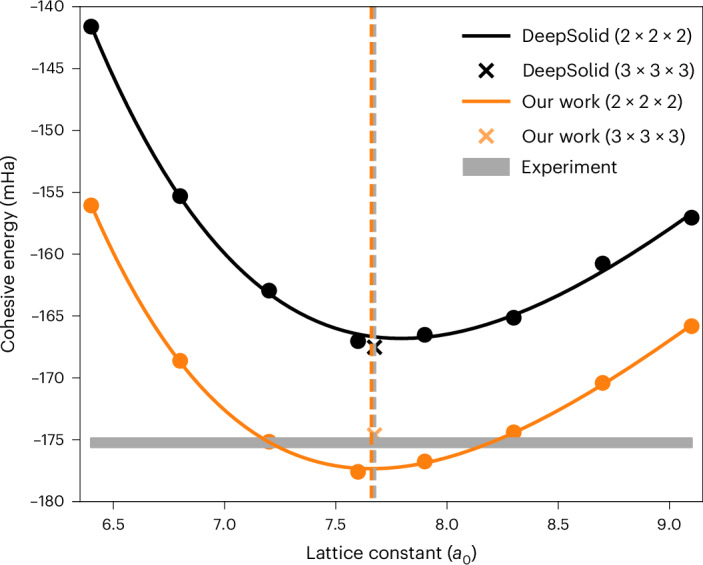
Energy–volume curve of LiH per primitive cell. The curve is for a 2 × 2 × 2 supercell as calculated using DeepSolid^18^ and our transferable DL-VMC method. The DeepSolid results (black circles, with a Birch–Murnaghan fit represented as a black line) were obtained at a single twist, the *Γ*-point. Hartree–Fock corrections were applied, as discussed in ref. ^18^, and a ZPVE correction was added. Our results (orange circles, with a Birch–Murnaghan fit represented as an orange line) are twist averaged, using a 5 × 5 × 5 Monkhorst–Pack grid per lattice constant. Structure-factor-based corrections were applied, and a ZPVE correction was added. The gray bar indicates the experimental uncertainty^26^. The statistical error bars are too small to be visible on this scale and therefore have been omitted. The vertical dashed orange line indicates the equilibrium lattice constant as calculated from the Birch–Murnaghan fit to our data. The vertical dashed gray line indicates the experimental value of the equilibrium lattice constant^26^. The orange cross shows the twist-averaged cohesive energy of a 3 × 3 × 3 simulation cell, again using structure factor correction. This was obtained by transferring the network pretrained for the 2 × 2 × 2 system to a 3 × 3 × 3 supercell, using only 8,000 additional optimization steps. A 5 × 5 × 5 Monkhorst–Pack grid of twists was used. The black cross shows the result of DeepSolid’s 3 × 3 × 3 *Γ*-point calculation with a Hartree–Fock finite-size correction. Source data

We have also used the transferable DL-VMC ansatz to evaluate the energy–volume curve of LiH in the rock-salt crystal structure. As shown in Fig. 5 (see also Supplementary Section 7), we obtain the energy–volume curve by fitting a Birch–Murhaghan equation of state to the total energies of a 2 × 2 × 2 supercell at eight different lattice parameters. To reduce finite-size errors, the eight total energies are twist averaged using a 5 × 5 × 5 *Γ*-centered Monkhorst–Pack grid and include structure-factor-based finite-size corrections. For comparison, DeepSolid performed a *Γ*-point calculation only and estimated finite-size errors by converging a Hartree–Fock calculation with an increasingly dense twist grid^18^. To all results we add ZPVE taken from ref. ^26^, making the calculated cohesive energy less negative by approximately 8 mHa. The DeepSolid results by Li et al.^18^ took no account of the ZPVE, explaining the slight difference between our depiction of their results, shown in Fig. 5, and their original publication^18^.

We trained a single neural network wavefunction across 8 lattice constants and 10 symmetry-reduced twists, making 80 systems in total. By comparison, DeepSolid required a separate calculation for each geometry.

The Birch–Murnaghan fit gives an equilibrium lattice constant of 7.66(1)*a*_0_ (dotted orange line), which agrees well with the experimental value of 7.674(2)*a*_0_ (ref. ^26^). Our Birch–Murnaghan estimate of the cohesive energy of −177.3(1) mHa per primitive cell deviates from the experimental value of −175.3(4) mHa by −2.0(5) mHa. This marks an improvement over the DeepSolid results^18^ of −166.8(1) mHa, which differ from experiment by 8.5(5) mHa. Because we are able to optimize all systems at once, our results were obtained with roughly 5% of the compute required by DeepSolid, and the speed-up is evident in Fig. 3b. Similar improvements can be observed in the variance of the local energy (Supplementary Section 8).

Although we improve on the DeepSolid baseline, the cohesive energy might still be impacted by finite-size effects because of the small size of the 2 × 2 × 2 supercell used. To check this, we also studied a larger supercell containing 3 × 3 × 3 primitive unit cells. This 108-electron system is one of the largest to have been studied using neural wavefunctions so far. DeepSolid used 400,000 optimization steps to get a *Γ*-point estimate for the cohesive energy and overestimated the energy by around 7 mHa per primitive cell compared with the experimental results^18,26^. By contrast, we can exploit the transferability of our wavefunction and use the parameters obtained from pretraining on the 2 × 2 × 2 supercells as initialization for the much larger 3 × 3 × 3 supercell. Due to the good generalization of our ansatz, we are able to calculate the cohesive energy for the 3 × 3 × 3 supercell with only 8,000 additional optimization steps shared across ten different twists. Using twist averaging, a structure-factor correction and a ZPVE correction as before, we obtain a cohesive energy of −174.6 mHa per primitive cell, deviating from experiment by only 0.7(5) mHa per primitive cell. The magnitude of this deviation is close to the 0.4-mHa spread of experimental data obtained from different thermochemistry experiments^26^. Our twist-averaged 3 × 3 × 3 calculation required only ~2% of the computational resources used by DeepSolid for a single *Γ*-point calculation^18^.

Furthermore, we compared our approach to the case of pretraining on a single system and fine-tuning the pretrained wavefunction on the remaining systems with independent calculations (similar to DeepSolid) for a 2 × 2 × 2 supercell of LiH. This comparison confirms that the approach of training a single neural network wavefunction across different systems converges much faster then fine-tuning independent wavefunctions (Supplementary Fig. 7).

## Discussion

By training a single transferable wavefunction across system sizes, geometries and boundary conditions, our approach substantially reduces the computational cost of applying DL-VMC to solids. Combining this approach with other acceleration techniques—such as the efficient forward evaluation of the Laplacian by Li et al.^27^ or pseudo-potentials^28^—might enable the study of strongly correlated materials with DL-VMC. Our approach could also be extended to grand-canonical twist averaging^29^, in which the number of electrons in the supercell varies with the twist. Because our ansatz already supports a variable number of particles, this extension should be easy to incorporate.

Our approach shares many of the limitations of other DL-VMC methods, including the sensitivity with regard to MCMC initialization. A standard practice in DL-VMC is to assign each electron a spin and initialize it close to the nuclei at the beginning of the calculation. If the electrons are initialized in an anti-ferromagnetic pattern, that is, alternating the spins of neighboring atoms, but the ground state is ferromagnetic, as can be the case for the hydrogen chain when the interatomic separation is small, our approach tends to converge to local minima. FermiNet suffers from similar problems.

Another limitation arises from the allocation of compute budget between the multiple geometries or systems described by a single neural network. We allocate more compute during optimization to twists with a larger weight, which has a positive effect on twist-averaged results in general, because twists with higher contribution are converged to higher accuracy (Table 1). However for individual twists, when plotting, for example, the band structure (Fig. 4), not all twists are optimized to the same accuracy, potentially skewing results.

While this work demonstrates the transferability of a wavefunction across variations of a system (lattice constant, supercell size and twist), more research is needed to develop wavefunctions that reliably transfer to entirely new systems, such as different compositions or lattices. Prior work on molecules in the gas phase has shown that it is possible to pretrain a single wavefunction on a diverse set of molecules and transfer the results to new, unseen molecules^30^. There are several open challenges to applying this approach to solids: First, the effectiveness decreases when transferring the pretrained model to systems substantially larger than those in the pretraining set. This issue is particular problematic for solids, where finite-size scaling may often require transferability to large systems. Second, successful pretraining typically requires wavefunction optimization for a large, diverse set of systems. This is challenging for calculations of solids, which are inherently more costly than calculations for molecules owing to the need for supercells. In practice, while pretraining on hundreds of qualitatively different systems is achievable on a moderate compute budget for gas-phase molecules, this scale is currently out of reach for solids.

## Methods

### Notation

All vectors, matrices and tensors are denoted by bold letters, except for functions. We use lower-case indices *i*, *j* = 1, …, *n*_el_ for electron positions and upper-case indices *I*, *J* = 1, …, *N*_atoms_ for atom positions, where *n*_el_ and *N*_atoms_ are the numbers of electrons and atoms in the supercell. Orbitals are enumerated by the indices *μ* and *ν*, which range from 1 to *n*_el_. The position of the *i*th electron is ${{\boldsymbol{r}}}_{i}\in {{\mathbb{R}}}^{3}$. When *i* is not used as a subscript, it denotes the imaginary unit. By ${\mathbf{r}}=({{\mathbf{r}}}_{1},\ldots ,{{\mathbf{r}}}_{{n}_{{\rm{el}}}})$, we denote the 3*n*_el_-dimensional vector of all electron positions. Similarly, nuclear positions and charges are represented by ${\mathbf{R}}=({{\mathbf{R}}}_{1},\ldots ,{{\mathbf{R}}}_{{N}_{\rm{atoms}}}\!)$ and ${\mathbf{Z}}=({Z}_{1},\ldots ,{Z}_{{N}_{{\rm{atoms}}}})$. The matrix ${L}\in {{\mathbb{R}}}^{3\times 3}$ contains the supercell lattice vectors in its columns. The twist vector, which may always be reduced into the first Brillouin zone of the supercell, is denoted by **k**_s_. The dot product of two vectors **a** and **b** is written **a** ⋅ **b**, and by ⊙ we refer to the element-wise multiplication (Hadamard product).

### Deep-learning VMC

The time-independent Schrödinger equation for a solid takes the form

2 $$\begin{array}{r}\hat{H}\varPsi =E\varPsi ,\qquad \hat{H}=-\frac{1}{2}\mathop{\sum}\limits _{i}{\nabla }_{{{\mathbf{r}}}_{i}}^{2}+{\hat{V}}_{{\rm{Coulomb}}}\end{array}$$

with the Hamiltonian in the Born–Oppenheimer approximation and Coulomb potential ${\hat{V}}_{{\rm{Coulomb}}}$. A finite supercell is used to approximate the bulk solid, and the Coulomb potential is evaluated using the Ewald method, as described in refs. ^14,31^.

In this work, we are interested in finding the lowest eigenvalue of the Schrödinger equation—the ground-state energy, *E*_0_—and the corresponding energy eigenfunction. To find an approximate solution, one can reformulate the Schrödinger equation as a minimization problem using the Rayleigh–Ritz variational principle. Given an arbitrary anti-symmetric trial wavefunction, *Ψ*_**θ**_, with **θ** denoting, for example, the trainable parameters of a neural network, the best attainable approximation to the ground state may be found by minimizing the energy expectation value

3 $$L({\bf{\uptheta }})={{\mathbb{E}}}_{{\bf{r}} \sim | {\varPsi }_{{\bf{\uptheta }}}{| }^{2}}\left[\frac{\hat{H}{\varPsi }_{{\bf{\uptheta }}}}{{\varPsi }_{{\bf{\uptheta }}}}\right]\ge {E}_{0}$$

with respect to **θ**. An important constraint for the construction of the trial wavefunction arises from the Pauli exclusion principle, which states that the wavefunction must be antisymmetric with respect to the permutations of different electron coordinates^6^. As in previous work, we approximate the expectation value in equation (3) using Monte Carlo integration with samples drawn from the 3*n*_el_-dimensional probability density ∣*Ψ*_**θ**_**(r**)∣^2^ (refs. ^6,8^).

A list of all relevant hyperparameters can be found in Supplementary Table 2.

### Architecture

#### Overview

Our ansatz can be broken down into the computation of periodic input features, the computation of embeddings **e**_*iJ*_ for each electron–nucleus pair, the computation of correlated orbitals and the assembly of the final wavefunction *Ψ*_*θ*_ as a sum of Slater determinants. Each step serves a distinct purpose.

The input features enforce the periodic boundary conditions of the supercell. To capture correlation effects, we use a neural network to map single-electron coordinates to vectors in a latent space. These vectors, also known as embeddings, depend on the positions of all of the other electrons in a permutation equivariant way. Each embedding therefore contains information about the corresponding electron as well as its environment. The embeddings are subsequently mapped to many-electron orbitals as outlined below.

#### Ansatz

Our wavefunction ansatz is a sum of Slater determinants multiplied by a Jastrow factor,

4 $$\varPsi ({\mathbf{r}},{\mathbf{R}},{\mathbf{Z}},{{\mathbf{k}}}_{{\rm{s}}})={{e}\;}^{J({\mathbf{r}})}\mathop{\sum }\limits_{d=1}^{{n}_{\det }}\det {{\Phi }}_{d}({\mathbf{r}},{\mathbf{R}},{\mathbf{Z}},{{\mathbf{k}}}_{{\rm{s}}}).$$

The optimization is free to adjust the relative normalizations of the determinants in the unweighted sum, making it equivalent to a weighted sum of normalized determinants, as might be used in a configuration-interaction expansion. The Jastrow factor *e*^*J*(***r***)^ is node-less and follows the work of Hermann et al.^8^, while the determinant enforces the fermionic antisymmetry. Instead of using single-particle orbitals in the determinant, as in most quantum chemical approaches, we follow other neural wavefunction methods^6^ and promote every entry *Φ*_*d*,*iμ*_ in the orbital matrix *Φ*_*d*_ from a one-electron orbital, *ϕ*_*d*,*μ*_(**r**_*i*_), to a many-electron orbital, *Φ*_*d*,*iμ*_(**r**) (temporarily dropping the dependency on **R**, **Z** and **k**_s_ for the sake of brevity). The many-electron orbitals are permutation equivariant, such that applying a permutation *π* to the electron position vectors permutes the rows of *Φ*_*d*_ by *π*, that is, ${\varPhi }_{d,i\mu }({{\boldsymbol{r}}}_{\pi (1)},\ldots ,{{\mathbf{r}}}_{\pi ({n}_{{\rm{el}}})})={\varPhi }_{d,\pi (i)\mu }({{\mathbf{r}}}_{1},\ldots ,{{\mathbf{r}}}_{{n}_{{\rm{el}}}})$. This ensures that the determinant has the correct fermionic symmetry. Each entry is constructed as a linear combination of atom-centered functions with permutation equivariant dependencies on both electrons and atoms

5 $${\varPhi }_{d,i\mu }({\mathbf{r}},{\mathbf{R}},{\mathbf{Z}},{{\mathbf{k}}}_{{\rm{s}}})={{e}}^{i{{\mathbf{k}}}_{{\rm{s}}}\cdot {{\mathbf{r}}}_{i}}\mathop{\sum }\limits_{J=1}^{{N}_{{\rm{atoms}}}}{\varphi }_{d\mu }({{\mathbf{r}}}_{i},\{{\mathbf{r}}\},{{\mathbf{R}}}_{J},\{{\mathbf{R}}\}).$$

Here, {**r**} and {**R**} denote the (permutation invariant) set of electron and atom positions, respectively. The phase factor enforces the twisted boundary conditions. To construct the *φ*_*dμiJ*_ ≡ *φ*_*dμ*_(**r**_*i*_, {**r**}, **R**_*J*_, {**R**}) using a neural network, we use an adaptation of the recently proposed transferable atomic orbital ansatz^21,30^. The orbitals are written as the inner product of an electron–nuclear embedding ${{\boldsymbol{e}}}_{iJ}\in {{\mathbb{R}}}^{{n}_{{\rm{emb}}}}$ and an orbital embedding ${{\mathbf{W}}}_{d\mu J}\in {{\mathbb{C}}}^{{n}_{{\rm{emb}}}}$, multiplied by an exponential envelope ${\varphi }_{d\mu J}^{\,\text{env}\,}$,

6 $${\varphi }_{d\mu iJ}=({{\mathbf{W}}}_{d\mu J}\cdot {{\mathbf{e}}}_{iJ}){\varphi }_{d\mu J}^{\,\text{env}\,}({{\mathbf{r}}}_{i})$$

7 $${\varphi }_{d\mu J}^{\,\text{env}\,}({{\mathbf{r}}}_{i})={e}^{-{a}_{d\mu J}| | {{\mathrm{L}}}^{-1}{{\mathbf{r}}}_{iJ}| {| }^{{\rm{per}}}},$$

where *a*_*dμJ*_ is a learnable decay rate, **s**_*iJ*_ is the vector from nucleus *J* to electron *i*, expressed in the basis of the supercell lattice vectors, and ∣∣**s**_*iJ*_∣∣^per^ is the modulus of **s**_*iJ*_ in a periodic norm explained below. Both the orbital embedding **W**_*dμJ*_ and the decay length *a*_*dμJ*_ depend on the orbital *μ* and atom *J* and are different for each determinant *d*.

To obtain **W**_*dμJ*_ and *a*_*dμJ*_ in a transferable way, we do not parameterize them directly but represent them as functions of some orbital-specific descriptor ${\tilde{{\mathbf{c}}}}_{\mu J}\in {{\mathbb{R}}}^{{d}_{{\rm{orb}}}}$:

8 $${{\mathbf{W}}}_{d\mu J}={f}_{d}^{\,\text{W}\,}\left({\tilde{{\mathbf{c}}}}_{\mu J}\right),\quad \quad \quad {a}_{d\mu J}={f}_{d}^{\;\,\text{a}\,}\left({\tilde{{\mathbf{c}}}}_{\mu J}\right),$$

with ${f}^{\;{\rm{W}}}:{{\mathbb{R}}}^{{d}_{{\rm{orb}}}}\to {{\mathbb{C}}}^{{n}_{\det }\times {d}_{{\rm{emb}}}}$ and ${f}^{\;{\rm{a}}}:{{\mathbb{R}}}^{{d}_{{\rm{orb}}}}\to {{\mathbb{R}}}^{{n}_{\det }}$ denoting simple multilayer perceptrons. The orbital embedding includes information about single-particle orbitals of the system calculated with a mean-field method, which is key for the transferability of the ansatz. The inputs are the orbital features ${\tilde{{\mathbf{c}}}}_{\mu J}\in {{\mathbb{R}}}^{{d}_{{\rm{orb}}}}$, which are concatenations of the expansion coefficients of the localized mean-field orbitals in an atom-centered basis set, the twist **k**_s_, the mean position of orbital *μ* and the position of atom *J*, with a combined dimensionality of *d*_orb_. While all parameters and intermediate computations of our network are real-valued, the last layer of *f*^W^ is complex-valued to allow the network to represent complex-valued wavefunctions.

An important difference with respect to previous neural network-based wavefunctions is the use of electron–nuclear embeddings **e**_*iJ*_, which describe the interaction between electron *i* and nucleus *J*. Other architectures such FermiNet, but also the more closely related transferable atomic orbital ansatz^21^, use embeddings to represent the interactions of a single electron *i* with all nuclei instead. However, when the embeddings are both invariant under permutation of nuclei (which we require for efficient transferability) and invariant under translation of particles by a supercell lattice vector (which we require to enforce boundary conditions), they become periodic on the primitive lattice (Supplementary Section 4), not just the supercell lattice. This is too restrictive to represent correlation beyond a single primitive cell. We therefore opt to use electron–nucleus embeddings that are equivariant under permutation of nuclei at some additional computational cost explained in Supplementary Section 4.

#### Input

We require our representation of the difference vectors **r**_*ij*_ = **r**_*i*_ − **r**_*j*_, **r**_*iI*_ = **r**_*i*_ − **R**_*I*_ and **r**_*IJ*_ = **r**_*I*_ − **R**_*J*_ to be periodic with respect to the supercell lattice. This is accomplished using the approach introduced by Cassella et al.^14^. The first step is to transform the coordinates into supercell fractional coordinates with **s**_*ij*_ = *L*^−1^**r**_*ij*_, **s**_*iI*_ = *L*^−1^**r**_*iI*_ and **s**_*IJ*_ = *L*^−1^**r**_*IJ*_. Periodic versions of the difference vectors are then obtained by applying sine and cosine element-wise,

9 $$\omega ({\mathbf{s}}):= [\sin (2\pi {\mathbf{s}}),\cos (2\pi {\mathbf{s}})],\qquad \omega :{{\mathbb{R}}}^{3}\to {{\mathbb{R}}}^{6},$$

10 $${{\mathbf{x}}}_{ij}:= \omega ({{\mathbf{s}}}_{ij}),\quad {{\mathbf{x}}}_{iJ}:= \omega ({{\mathbf{s}}}_{iJ}),\quad {{\mathbf{x}}}_{IJ}:= \omega ({{\mathbf{s}}}_{IJ}),$$

where square brackets denote the concatenation operator. For the distance, we use the periodic norm

11 $${\left(| | {\mathbf{s}}| {| }^{{\rm{per}}}\right)}^{2}=\mathop{\sum }\limits_{l,p=1}^{3}\left(\left(1-\cos (2\pi {s}_{l})\right){A}_{lp}\left(1-\cos (2\pi {s}_{p})\right)+\sin (2\pi {s}_{l}){A}_{lp}\sin (2\pi {s}_{p})\right)$$

for a vector ${\mathbf{s}}\in {{\mathbb{R}}}^{3}$ with the lattice metric *A* ≔ *LL*^*T*^. This norm is used to define the periodic distance features

12 $${x}_{ij}=| | {{\mathbf{s}}}_{ij}| {| }^{{\rm{per}}},\quad {x}_{iJ}=| | {{\mathbf{s}}}_{iJ}| {| }^{{\rm{per}}},\quad {x}_{IJ}=| | {{\mathbf{s}}}_{IJ}| {| }^{{\rm{per}}}.$$

#### Embedding

The periodic input features are used to generate high-dimensional embeddings **e**_*iJ*_ for the construction of the orbital matrix. The following embedding is a slight adaption of the appraoch used in the recently proposed Moon architecture^32^. We start by aggregating the electron–electron features into message vectors ${{\mathbf{m}}}_{i}^{0}$ for each electron *i*

13 $${{\mathbf{m}}}_{i}^{0}=\mathop{\sum }\limits_{j=1}^{{n}_{{\rm{el}}}}{\varGamma }^{{\rm{e}}\text{-}{\rm{e}}}({x}_{ij},\,{{\mathbf{x}}}_{ij})\odot \sigma \left({{W}}^{{\rm{m}}}{\tilde{{\mathbf{x}}}}_{ij}+{{\mathbf{b}}}^{{\rm{m}}}\right),$$

and compute the initial electron embeddings ${{\mathbf{h}}}_{i}^{0}$ as a trainable function of these messages

14 $${{\mathbf{h}}}_{i}^{0}=\sigma \left({{W}}^{0}{{\mathbf{m}}}_{i}^{0}+{{\mathbf{b}}}^{0}\right).$$

The matrices *W*^m^ and *W*^0^ and vectors **b**^m^ and **b**^0^ are trainable parameters, *σ* is an activation function that is applied elementwise and ⊙ denotes the elementwise product. The filter function *Γ*^e-e^

15 $${\varGamma }^{{\rm{e}}\text{-}{\rm{e}}}({x}_{ij},\,{{\mathbf{x}}}_{ij})=\sigma \left({{W}}^{{\rm{env}}}{{\mathbf{x}}}_{ij}+{\mathbf{b}}\right)\odot \exp \left(-{x}_{ij}^{2}{\bf{\upalpha }}\right),$$

ensures an exponential decay with a trainable vector of length scales **α** and a trainable matrix *W*^env^. Furthermore, the input features ${\tilde{{\mathbf{x}}}}_{ij}=[{x}_{ij},{{\mathbf{x}}}_{ij},{{\mathbf{k}}}_{{\rm{s}}}]$ make the embedding twist dependent to allow for better transferability across twists.

To initialize the atomic features, we first one-hot encode the nuclear charges **Z** into a matrix $\tilde{{H}}\in {{\mathbb{R}}}^{{N}_{{\rm{atoms}}}\times {n}_{{\rm{species}}}}$. With one-hot encoding we refer to the common machine-learning practice of encoding categorical data (in this case, the type of atom), using a vector that is zero everywhere, except in the one dimension corresponding to the category it encodes. We then initialize the atom embeddings ${{\mathbf{H}}}_{I}^{0}$ analogously to the electron embeddings, by aggregating atom–atom features for each atom *I*

16 $${{\mathbf{H}}}_{I}^{0}=\mathop{\sum }\limits_{J=1}^{{N}_{{\rm{atoms}}}}{\varGamma }^{{\rm{a}}\text{-}{\rm{a}}}({x}_{IJ},\,{{\mathbf{x}}}_{IJ})\odot \sigma \left({{W}}^{{\rm{a}}}{\tilde{{\mathbf{H}}}}_{J}+{{\mathbf{b}}}^{{\rm{a}}}\right),$$

using a trainable weight matrix *W*^a^ and bias vector **b**^a^. We then incorporate electron–atom information by contracting across all electrons

17 $${{\mathbf{H}}}_{I}^{1}=\mathop{\sum }\limits_{i=1}^{{n}_{{\rm{el}}}}{{\mathbf{e}}}_{iI}^{0}\odot \left({{W}}^{{\rm{e}}\text{-}{\rm{a}}}\,{\varGamma }^{{\rm{e}}\text{-}{\rm{a}}}({x}_{iI},\,{{\mathbf{x}}}_{iI})\right)$$

18 $${{\mathbf{e}}}_{iI}^{0}=\sigma \left({{\mathbf{h}}}_{i}^{0}+{{\mathbf{H}}}_{I}^{0}+{{W}}^{{\rm{edge}}}{\tilde{{\mathbf{x}}}}_{iI}+{{\mathbf{b}}}^{{\rm{edge}}}\right),$$

with ${\tilde{{\mathbf{x}}}}_{iI}=[{x}_{iI},{{\mathbf{x}}}_{iI},{{\mathbf{k}}}_{{\rm{s}}}]$ and trainable matrices *W*^e-a^, **W**^edge^ and bias **b**^edge^. Subsequently, the atom embeddings are updated with *L* dense layers

19 $${{\mathbf{H}}}_{I}^{l+1}=\sigma \left({{W}}^{l}{{\mathbf{H}}}_{I}^{l}+{{\mathbf{b}}}^{l}\right)+{{\mathbf{H}}}_{I}^{l},$$

to finally diffuse them to electron-atom embeddings **e**_*iI*_ of the form

20 $${{\mathbf{e}}}_{iI}=\sigma \left({{W}}^{{\text{out}}_{1}}{{\mathbf{e}}}_{iI}^{0}+{{\mathbf{H}}}_{I}^{L}+{{W}}^{{\text{out}}_{2}}{{\mathbf{h}}}_{i}^{0}+{{\mathbf{b}}}^{{\rm{out}}}\right)\odot \left({{W}}^{{\text{out}}_{3}}\,{\varGamma }^{{\rm{out}}}({x}_{iI},\,{{\mathbf{x}}}_{iI})\right).$$

with trainable matrix ${{W}}^{{\text{out}}_{1}},{{W}}^{{\text{out}}_{2}},{{W}}^{{\text{out}}_{3}}$ and trainable bias vector **b**^out^. For the sake of simplicity, we omitted the spin dependence in this presentation of the different embedding stages. Compared with the original Moon embedding^32^, we use separate filters *Γ* for the intermediate layers and the output layer, include the twist as input feature and omit the final aggregation step from electron–ion embeddings **e**_*iI*_ to electron embeddings **e**_*i*_.

#### Orbitals

The orbital features ${\tilde{{\mathbf{c}}}}_{\mu J}$ are a concatenation of four different types of features. First, as proposed by Scherbela et al.^21^, we rely on mean-field coefficients from a Hartree–Fock calculation. The mean-field orbitals *ϕ*_*μ*_ are localized as described in ‘Orbital localization’ and expanded in periodic, atom-centered, basis functions *b*_*η*_

21 $${\phi }_{\mu }({{\mathbf{r}}}_{i})=\mathop{\sum }\limits_{I=1}^{{N}_{{\rm{atoms}}}}\mathop{\sum }\limits_{\eta =1}^{{n}_{{\rm{b}}}}{c}_{I\mu ,\eta }\ {b}_{\eta }({{\mathbf{r}}}_{i}-{{\mathbf{R}}}_{I}),$$

where *n*_b_ represents the per-atom basis set size of the Hartree–Fock calculation. We use a periodic version of the cc-pVDZ basis set^33^ and find no strong dependence of our results on the basis set used (Supplementary Fig. 5). In addition, we include relative atom positions ${\tilde{{\mathbf{R}}}}_{I}$

22 $${\tilde{{\mathbf{R}}}}_{I}={{\mathbf{R}}}_{I}-\frac{\mathop{\sum}\nolimits_{J=1}^{{N}_{{\rm{atoms}}}}{{\mathbf{R}}}_{J}{Z}_{J}}{\mathop{\sum}\nolimits_{K=1}^{{N}_{{\rm{atoms}}}}{Z}_{K}}$$

and analogously relative orbital positions ${\tilde{{\mathbf{R}}}}_{\mu }^{\,\text{orb}\,}$

23 $${\tilde{{\mathbf{R}}}}_{\mu }^{\,\text{orb}\,}={{\mathbf{R}}}_{\mu }^{\,\text{orb}\,}-\frac{\mathop{\sum}\nolimits_{J=1}^{{N}_{{\rm{atoms}}}}{{\mathbf{R}}}_{J}{Z}_{J}}{\mathop{\sum}\nolimits_{K=1}^{{N}_{{\rm{atoms}}}}{Z}_{K}},$$

where ${{\mathbf{R}}}_{\mu }^{\,\text{orb}\,}$ is the position of the localized orbital *μ* as outlined in ‘Orbital localization’. This allows the network to differentiate between different atoms and orbitals within the supercell. As a final feature, we include the twist of the system

24 $${\tilde{{\mathbf{k}}}}_{I}^{\,\text{s}\,}=[{{\mathbf{k}}}_{{\rm{s}}},\,\sin ({{\mathbf{R}}}_{I}\cdot {{\mathbf{k}}}_{{\rm{s}}}),\cos ({{\mathbf{R}}}_{I}\cdot {{\mathbf{k}}}_{{\rm{s}}})]\in {{\mathbb{R}}}^{5}.$$

The final orbital features ${\tilde{{\mathbf{c}}}}_{I\mu }$ are obtained as a concatenation

25 $${\tilde{{\mathbf{c}}}}_{I\mu }=[{{\mathbf{c}}}_{I\mu },\,{\tilde{{\mathbf{R}}}}_{I},\,{\tilde{{\mathbf{R}}}}_{\mu }^{\,\text{orb}\,},\,{\tilde{{\mathbf{k}}}}_{I}^{\,\text{s}\,}]\in {{\mathbb{R}}}^{{d}_{{\rm{orb}}}},$$

where *d*_orb_ = *n*_*b*_ + 11, resulting from the concatenation of the *n*_*b*_ basis coefficient features, 3 atom position features, 3 orbital position features and 5 twist features.

### Sampling

We use the Metropolis Hastings algorithm^34^ to draw samples **r** from our unnormalized density ∣*Ψ*_**θ**_∣^2^. We use Gaussian all-electron proposals **r**^prop^ of the form

26 $${{\mathbf{r}}}^{{\rm{prop}}}={\mathbf{r}}+s{\bf{\updelta }},$$

where **δ** is drawn from a 3*n*_el_-dimensional standard normal distribution. We continuously adjust the stepsize *s* to obtain a mean acceptance probability of approximately 50%. Empirically, we find no strong dependence of autocorrelations on this acceptance target, as long as it is roughly between 30% and 70%. While it can be shown that under simplifying assumptions 23% is the optimal acceptance rate^35^, we do not find this to be optimal in practice. Performance is more strongly impacted by too small acceptance rates, and thus, we opt for the larger ~50%.

When calculating properties of the hydrogen chain for different lattice constants *R*, special care must be given to the treatment of spins. The hydrogen chain has two phases with different arrangements of spins. In the insulating phase at large lattice constant, the ground state is antiferromagnetic, that is, neighboring spins prefer to be aligned antiparallel. In the metallic phase at small lattice constant, this antiferromagnetic ordering decreases and the system may even show ferromagnetic domains^3^. Moving between these two configurations is difficult using local Monte Carlo updates as given by equation (26), so we modify our Metropolis Hastings proposal function. In addition to moving electrons in real space, we occasionally propose moves that swap the positions of two electrons with opposite spin. To avoid biasing our sampling toward either spin configuration, we initialize half our Monte Carlo walkers in the antiferromagnetic configuration (neighboring electrons having opposite spin) and half our Monte Carlo walkers in a ferromagnetic configuration (all spin-up electrons in one half of the chain and all spin-down electrons in the other half). We found that, on the contrary, initializing all walkers in the antiferromagnetic configuration (as might be indicated, for example, by a mean-field calculation) can cause the optimization to fall into local energy minima during wavefunction optimization.

When optimizing a transferable wavefunction across multiple systems, we must also sample these systems during training. To simplify implementation, we sample only a single system per gradient step. We choose this system randomly, with its probability being either proportional to the systems weight (in the case of twist averaging) or proportional to the variance per electron.

### Complex KFAC

We use the Kronecker factored approximate curvature (KFAC) method^36^ to optimize the trainable parameters of our ansatz. KFAC uses the Fisher information matrix as a metric in the space of wavefunction parameters. For real wavefunctions, the Fisher matrix is equivalent to the preconditioner used in the stochastic reconfiguration method ^6^, but this is not the case for complex wavefunctions. Instead, the Fubini–Study metric should be used, given by

27 $${F}_{ij}={\rm{Re}}\left\{\left\langle {\frac{\partial \ln \uppsi }{\partial {\theta }_{i}}}^{* }\frac{\partial \ln \uppsi }{\partial {\theta }_{j}}\right\rangle \right\}$$

Writing the complex wavefunction in polar form, *ψ* = *ρe*^*iϕ*^, this becomes

28 $$F=\left\langle \frac{\partial \ln \rho }{\partial {\theta }_{i}}\frac{\partial \ln \rho }{\partial {\theta }_{j}}+\frac{\partial \phi }{\partial {\theta }_{i}}\frac{\partial \phi }{\partial {\theta }_{j}}\right\rangle ,$$

where the first term is the Fisher information matrix and the second term is the new contribution due to the phase of the wavefunction. The second term is zero if the phase is a global constant, such as for a purely real-valued wavefunction. For our wavefunction, the phase is generally nonzero, due to the complex-valued orbitals and the phase factor introduced to enforce twist-averaged boundary conditions.

### Orbital localization

To obtain orbital features that generalize well across system sizes, we do not use the canonical mean-field coefficients **c** as network inputs. Rather, we use the coefficients **c**^loc^ of maximally localized Wannier orbitals computed from **c**. We follow the procedure of ref. ^37^ to find a unitary rotation *U* within the subspace spanned by the occupied orbitals. Given a set of mean-field orbitals *ϕ*_*μ*_(**r**), *μ* = 1, …, *n*_el_, expanded in periodic, atom-centered basis functions *b*_*Iη*_(**r**), *I* = 1, …, *N*_atoms_, *η* = 1, …, *n*_b_, as described in ‘Architecture’, we compute the complex polarization matrix

29 $${\chi }_{\alpha ,\nu \mu }=\int\,{\phi }_{\nu }^{* }({\mathbf{r}}){e}^{i{{\mathbf{r}}}^{T}{{\mathbf{G}}}_{\alpha }}{\phi }_{\mu }({\mathbf{r}})\,\text{d}\,{\mathbf{r}},\quad \chi \in {{\mathbb{C}}}^{3\times {n}_{{\rm{orb}}}\times {n}_{{\rm{orb}}}}$$

where *G* = 2π*L*^−*T*^ is the matrix of reciprocal lattice vectors. Given a unitary transformation ${\mathbf{U}}\in {{\mathbb{C}}}^{{n}_{{\rm{orb}}}\times {n}_{{\rm{orb}}}}$, the transformed polarization matrix ${\hat{\upchi }}$ and the corresponding localization loss ${\mathcal{L}}$ are given by

30 $${{\bf{\Omega }}}_{\alpha \mu }={\hat{{\bf{\upchi }}}}_{\alpha ,\mu \mu }={\left({{U}}^{\dagger }{\chi }_{\alpha }{U}\right)}_{\mu \mu }$$

31 $${\mathcal{L}}({U})=-\parallel {\boldsymbol{\Omega }}({U}){\parallel }_{2}^{2},$$

where ∥⋅∥_2_ denotes the L_2_ norm. To facilitate unconstrained optimization, we parameterize the unitary matrix *U* as the complex matrix exponential of a symmetrized, unconstrained complex matrix *A*:

32 $${{U}}={e}^{\frac{i}{2}({\mathbf{A}}+{{\mathbf{A}}}^{\dagger })}.$$

We obtain the optimal *U*^loc^, and corresponding orbital coefficients **c**^loc^ via gradient-based optimization

33 $${{{U}}}^{{\rm{loc}}}={{\rm{argmin}}}_{{{U}}}{\mathcal{L}}({{U}}),\qquad {c}_{I\eta ,\mu }^{\,\text{loc}}=\sum _{m}{c}_{I\eta ,\nu }{U}_{\nu \mu }^{\text{loc}\,},$$

using the Adam ^38^ optimizer. For orthorombic supercells, the position of the Wannier center ${{\mathbf{R}}}_{\mu }^{\,\text{orb}\,}$ of the localized orbital *μ* can be inferred from the localized polarization matrix $\hat{{\bf{\upchi }}}$ as

34 $${R}_{l\alpha }^{\,\text{orb}\,}=-\frac{{{L}}_{\alpha \alpha }}{2\pi }\,\text{Im}\,\log {\hat{{\bf{\upchi }}}}_{\mu \mu }^{\alpha },\qquad \alpha =1\ldots 3,\ \ \ \mu =1\ldots {n}_{{\rm{orb}}}.$$

For other supercells, we follow the generalization given in ref. ^37^.

### Observables and postprocessing

#### TABC

In a finite system, there are finite-size errors related to both the artificial constraint of periodicity in the supercell and the lack of correlations of longer range than the supercell. The effects of the former on the single-particle contributions to the Hamiltonian, namely the kinetic energy, the Hartree energy and the electron–ion interaction, can be reduced using TABC^20,25^. Twisted boundary conditions require that the wavefunction obeys

35 $$\varPsi ({{\mathbf{r}}}_{1},\ldots ,{{\mathbf{r}}}_{i}+{{\mathbf{L}}}_{\alpha },\ldots ,{{\mathbf{r}}}_{N})={e}^{i{\mathbf{k}}\cdot {{\mathbf{L}}}_{\alpha }}\varPsi ({{\mathbf{r}}}_{1},\ldots ,{{\mathbf{r}}}_{i},\ldots ,{{\mathbf{r}}}_{N}),$$

where **L**_*α*_ is the *α*th supercell lattice vector. Equation (35) is enforced by adding a position-dependent phase ${e}^{i{{\mathbf{k}}}_{s}\cdot {{\mathbf{r}}}_{i}}$ for each electron in the transferable atomic orbitals. as seen in equation (5). To obtain twist-averaged observables, we compute observables across a grid of twists **k**_s_ spanning the first Brillouin zone and average the results.

#### Structure factor correction

To handle finite-size errors in the Ewald energy, we use the finite-size corrections proposed by ref. ^25^. Writing the Ewald energy in terms of Fourier series, we get

36 $$\left\langle \hat{{V}_{{\mathrm{E}}}}\right\rangle =\frac{N}{2}\left\{{v}_{{\mathrm{M}}}+\frac{1}{\varOmega }\sum _{{{\mathbf{G}}}_{{\mathrm{s}}}\ne 0}{v}_{{\mathrm{E}}}({G}_{{\mathrm{s}}})[S({{\mathbf{G}}}_{{\mathrm{s}}})-1]\right\}+\frac{1}{2\varOmega }\sum _{{{\mathbf{G}}}_{{\mathrm{p}}}\ne 0}{v}_{{\mathrm{E}}}({G}_{{\mathrm{p}}})\rho ({{\mathbf{G}}}_{{\mathrm{p}}}){\rho }^{* }({{\mathbf{G}}}_{{\mathrm{p}}}).$$

Here, *v*_M_ is the Madelung energy, *Ω* is the supercell volume, *v*_E_(**k**) = 4*π*/*k*^2^ is the Fourier transform of the Coulomb interaction, and **G**_s_ (**G**_p_) is a simulation (primitive) cell reciprocal lattice vector. The translationally averaged structure factor *S*(**G**_p_) is defined by

37 $$S({\mathbf{G}}_{\mathrm{s}})=\frac{1}{N}\left[\langle {\;\hat{\rho}} ({\mathbf{G}}_{\mathrm{s}}){\hat{\rho}}^{*}({\mathbf{G}}_{\mathrm{s}})\rangle -\langle {\;\hat{\rho}} ({\mathbf{G}}_{\mathrm{s}})\rangle \langle {\;\hat{\rho}}^{*}({\mathbf{G}}_{\mathrm{s}})\rangle \right],$$

where $\hat{\rho }({{\mathbf{G}}}_{{\mathrm{s}}})=\sum _{i}\exp \left(-i{{\mathbf{G}}}_{{\mathrm{s}}}\cdot {{\mathbf{r}}}_{i}\right)$ is the Fourier representation of the operator for the electron density. The structure factor converges fairly rapidly with supercell size, so we can assume that *S*_*Ω*_(**k**) ≈ *S*_*∞*_(**k**). In this limit, the largest contribution to the error is the omission of the *G*_s_ = 0 term in the first sum. In cubic systems, we have *S*(**k**) ∝ *ηk*^2^ + *O*(*k*^4^), with odd terms missing due to inversion symmetry, and the **k** → 0 limit of *S*(**k**)*v*_E_(*k*) is well defined. As such, to a first approximation, the Ewald finite-size error is given by

38 $$\Delta {V}_{{\mathrm{E}}}\approx \frac{N}{2\varOmega }\mathop{\lim }\limits_{k\to 0}{v}_{{\mathrm{E}}}(k)S({\mathbf{k}})=\frac{4\pi N}{2\varOmega }\mathop{\lim }\limits_{k\to 0}\frac{S({\mathbf{k}})}{{k}^{2}}.$$

Sampling *S*(**G**_s_) at supercell reciprocal lattice vectors **G**_s_, we approximate the limit *k* → 0 by fitting the function

39 $$S(k)\approx f(k)=1-{e}^{-{a}_{0}{k}^{2}-{a}_{1}{k}^{4}},$$

with *a*_0_ and *a*_1_ greater than zero. The form of the fit ensures that *S*(*k*) has the correct *k*^2^ behavior at small *k* and that $\mathop{\lim }\limits_{k\to \infty }S({\mathbf{k}})=1$. The finite-size correction Δ*V*_E_ is given by Δ*V*_E_ ≈ 4π*Na*_0_/2*Ω*.

#### ZPVE

To estimate the ZPVE contribution for graphene, we obtained the phonon density of states *D*(*ω*) calculated within DFT using the Perdew–Burke–Ernzerhof functional^2^ from ref. ^39^. The ZPVE energy per primitive cell, *E*_ZPVE_, is then given as

40 $${E}_{\rm{ZPVE}}=\frac{3{N}_{{\rm{atoms}}}^{\text{prim}\,}}{\int \! D(\omega )\,\text{d}\,\omega }\int \! D(\omega )\frac{1}{2}\hslash \omega \,\text{d}\,\omega ,$$

where ${N}_{\,\text{atoms}}^{\text{prim}\,}=2$ is the number of atoms per primitive unit cell of graphene. This yields a ZPVE of 12.8 mHa per primitive cell for graphene. For LiH, we use ZPVE data published in ref. ^26^.

## Supplementary information

Supplementary Information Supplementary Figs. 1–7 and Tables 1–4.

## Source data

Source Data Fig. 2 Data points per method and number of atoms; data points per method and geometry.

Source Data Fig. 3 Data points over optimization steps per twist for graphene; data points over optimization steps per twist for LiH.

Source Data Fig. 4 Data points per twist.

Source Data Fig. 5 Data point per geometry and twist for LiH.

## Data Availability

All data, including geometries, configurations and the figure source data, are available via GitHub at https://github.com/mdsunivie/deeperwin and via Zenodo at 10.5281/zenodo.16084892 (ref. ^40^). Source data are provided with this paper.

## Code availability

All code is available via GitHub at https://github.com/mdsunivie/deeperwin and via Zenodo at 10.5281/zenodo.16084892 (ref. ^40^).
