# Supplementary material for: Transferable neural wavefunctions for solids
Source: Nat Comput Sci. 2025 Oct 22;5(12):1147–57. doi: 10.1038/s43588-025-00872-z (PMC12727494; doi:10.1038/s43588-025-00872-z)
Supplement: Supplementary file 1 — Supplementary Figs. 1–7 and Tables 1–4. [file 43588_2025_872_MOESM1_ESM.pdf]

nature computational science

Supplementary information

https://doi.org/10.1038/s43588-025-00872-z

Transferable neural wavefunctions for solids

In the format provided by the authors and unedited

1 Properties of the H-chain

Equilibrium geometry Using our transferable wavefunction we can efficiently compute potential energy surfaces. For the hydrogen chain, for example, we can determine the equilibrium distance between the H atoms as a function of supercell size by optimizing a single wavefunction across the number of atoms N_{atom} per supercell, atom separation R and twist k. Supplementary Figure 1 shows the potential energy surface and resulting equilibrium distance, R_0, obtained by training a single wavefunction across 5 supercell sizes, 4 atom separations and 3 twists. We only find a weak dependence of the equilibrium geometry on the supercell size. Note that these equally-spaced equilibrium geometries are saddle points because the H chain undergoes dimerization, leading to alternating shorter and larger bond lengths [1].

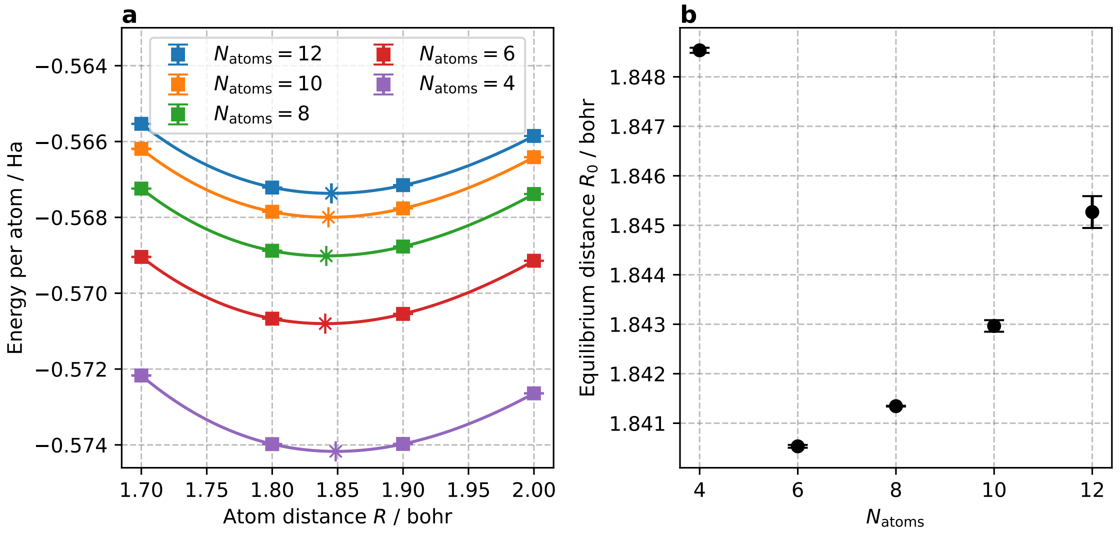

Supplementary Figure 1: **Potential energy surface of the H chain**: **a**: Energy per atom for supercells with N_{atom}=2-12 atoms. Square markers are energies obtained from the transferable wavefunction. Crosses denote the equilibrium separation R_0 obtained from a Morse-potential fit (solid line). **b**: Equilibrium distance R_0 as a function of the number of atoms per supercell. The error bars represent Monte Carlo uncertainty.

Twist-resolved polarization $|z|$ Supplementary Figure 2 depicts the modulus of the complex polarization $|z|$ as a function of twist. This complements Fig. 2b in the main text, which depicts the twist-averaged $|z|$ as a function of R. At large inter-atomic separations R, we find that the chain is always insulating ($|z| > 0$), independent of the twist vector k; at small separations the chain is always metallic ($|z|$=0). At intermediate values of R there is a strong twist dependence, highlighting the need for twist-averaged calculations.

Critical atom separation for metal insulator transition To determine the critical atom separation R_{crit} for the MIT at $N = 40$ in Fig. 2b, we fit a models of the following form

$$|z(R)| = \begin{cases} 1 - e^{-\alpha(R-R_{\text{crit}})-\beta(R-R_{\text{crit}})}, & \text{if } R > R_{\text{crit}} \\ 0, & \text{if } R \leq R_{\text{crit}}, \end{cases} \tag{1}$$

with free parameters $\alpha, \beta, R_{\text{crit}} > 0$. We obtain $R_{\text{crit}} = 1.74a_0$ and $R_{\text{crit}} = 1.61a_0$ for the AFQMC and DMC data from [2] and $R_{\text{crit}} = 1.32a_0$ using our approach.

Spin correlations In addition to energies and polarizations, other properties such as electron densities and spin correlations can be extracted from the wavefunction. Supplementary Figure 3 depicts the probabilities of finding pairs of spin-parallel and spin-antiparallel electrons separated

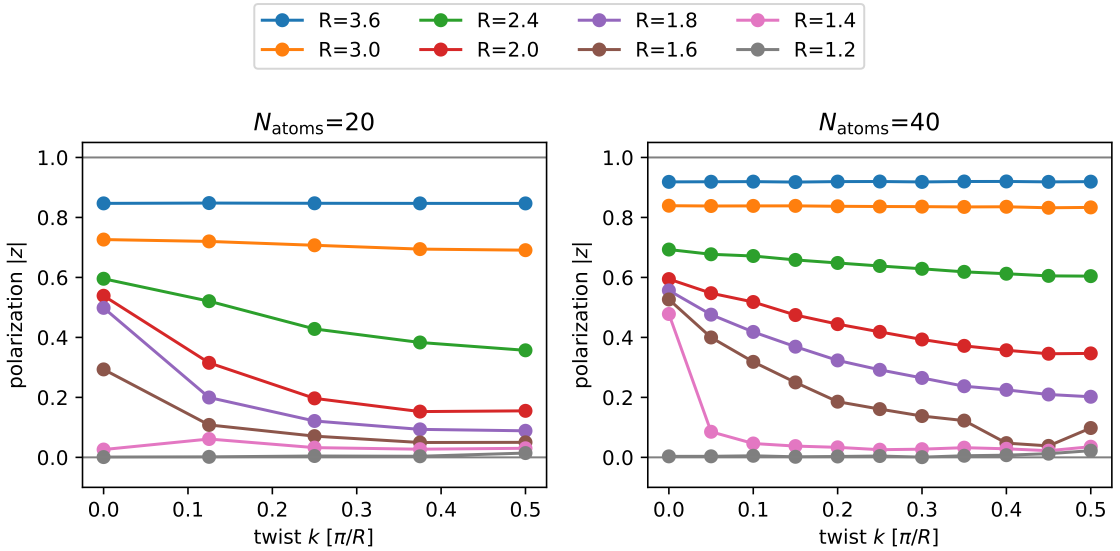

Supplementary Figure 2: **Twist dependence of complex polarization $|z|$ for the hydrogen chain**

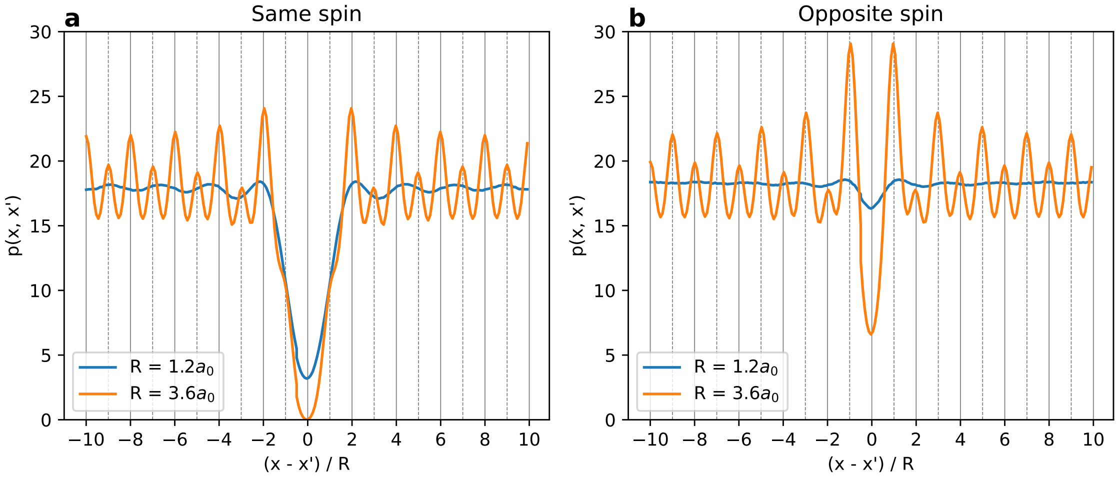

Supplementary Figure 3: **Pair correlations in H_{20}** The probability density $p(x, x')$ of finding pairs of electrons at positions x and x' as a function of their separation. **a**: pairs of electrons with the same spin, **b**: pairs of electrons with the opposite spin

by a distance $x - x'$ in 20-atom H chains with two different inter-atomic separations R. At large separation, there is a strong anti-ferromagnetic ordering, *i.e.*, there is a high probability of finding a pair of electrons with the same spin separated by a distance $|x - x'| = 2R, 4R, \ldots, 10R$, and a high probability of finding electrons of opposing spin at $|x - x'| = R, 3R, \ldots, 9R$. At shorter separation, the antiferromagnetic long-range order disappears. This is consistent with Ref. [2], which also reported a decrease in the strength of the anti-ferromagnetic correlations with decreasing inter-atomic separation. For electrons with identical spin there is a minimum at $x = x'$ due to Pauli exclusion. The probability is not zero, because we average across the y and z coordinates transversal to the chain. Therefore at $x = x'$, pairs of electrons may still differ in their y and z coordinate, yielding a non-zero probability of finding a pair at $x = x'$.

2 Cohesive energies of graphene

Experimental cohesive energies for graphene are obtained from thermochemistry data for graphite [3] corrected for the small inter-layer binding energy of 3.5 mHa obtained using the Random Phase Approximation [4].

Supplementary Table 1: **Cohesive energies of graphene** in Hartrees. The table compares the twist-averaged cohesive energy per primitive cell with experimental results, showing the effect of increasing the size of the twist grid from 3×3 to 12×12. For the calculation of the cohesive energy we follow Li et al. [5] and take as the energy of a single carbon atom E= -37.84471 Ha [6]. All results include a structure-factor-based finite-size correction [7] and ZPVE (see Sec. "Observables and post-processing"). The experimental results are based on Brewer [3] and Lebègue et al. [4].

	**Twist grid**	**2×2 supercell**	**3×3 supercell**	**Experiment**
DeepSolid	3×3	-0.5375	-	$-0.538(2)$
	12×12	-	-	
Our work	3×3	$-0.5413(2)$	$-0.5220(2)$	
	12×12	$-0.5451(2)$	$-0.5245(2)$	

3 Scaling of compute cost with system size

Computing the orbital matrix Φ_{ik} in our ansatz for each electron i and orbital k requires a sum over all nuclei J. Since the number of orbitals and electrons are equal to n_{el} and the number of nuclei N_{atoms} is in a worst-case also equal to n_{el}, materializing this matrix has a worst-case scaling of $\mathcal{O}({n_{\text{el}}}^3)$. This is in contrast to other approaches such as FermiNet, where this matrix is not given as a sum over nuclei and thus only scales as $\mathcal{O}({n_{\text{el}}}^2)$. Because scaling in the limit of $n_{\text{el}} \to \infty$ is in either case dominated by the evaluation of the determinant, which scales as $\mathcal{O}({n_{\text{el}}}^3)$, this does not impact the overall scaling of the method, but can lead to different empirical scaling. Supplementary Figure 4 depicts median run-time per optimization step for FermiNet (our implementation) and our approach. We compare timings on chains of Hydrogen atoms of increasing length and dimers of increasing nuclear charge. The former depicts the worst case for our method, the latter is close to the best case. All timings are obtained on 2 A100-GPUs using a batch-size of 512 and 8 determinants.

4 Limited expressiveness of electron-wise embeddings

Prior work has relied on embeddings $\boldsymbol{h}_i$ for each electron i to construct correlated orbitals. We show that requiring the following three reasonable symmetries already overly constrains which embeddings $\boldsymbol{h}_i$ (and consequently orbitals Φ) can be represented:

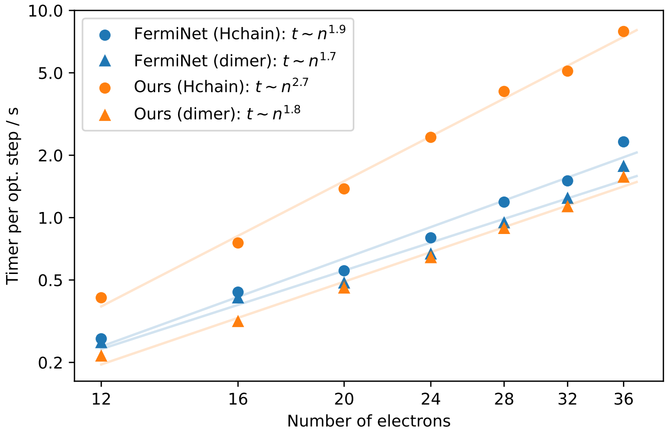

Supplementary Figure 4: **Scaling of computational cost**: Markers correspond to measured timings, lines correspond to least-square fits of power-laws, with the exponents denoted in the legend.

Invariance wrt. to continuous translation of *all* particles:

$$h(\boldsymbol{r}_1 + \boldsymbol{\delta}, \dots \boldsymbol{r}_{n_{\text{el}}} + \boldsymbol{\delta}, \boldsymbol{R}_1 + \boldsymbol{\delta}, \dots, \boldsymbol{R}_{N_{\text{atoms}}} + \boldsymbol{\delta}) = h(\boldsymbol{r}_1, \dots, \boldsymbol{r}_{n_{\text{el}}}, \boldsymbol{R}_1, \dots, \boldsymbol{R}_{N_{\text{atoms}}}). \tag{2}$$

Invariance wrt. to permutation of nuclei of same charge Z:

$$h(\boldsymbol{r}_1, \dots, \boldsymbol{r}_{n_{\text{el}}}, \boldsymbol{R}_1, \dots, \boldsymbol{R}_I, \dots \boldsymbol{R}_J, \dots \boldsymbol{R}_{N_{\text{atoms}}}) = h(\boldsymbol{r}_1, \dots, \boldsymbol{r}_{n_{\text{el}}}, \boldsymbol{R}_1, \dots, \boldsymbol{R}_J, \dots \boldsymbol{R}_I, \dots \boldsymbol{R}_{N_{\text{atoms}}}). \tag{3}$$

Invariance wrt. to translation of any particle by a supercell lattice vector $\boldsymbol{L}_{\text{sc}}$:

$$h(\boldsymbol{r}_1 + \boldsymbol{L}_{\text{sc}}, \dots, \boldsymbol{r}_{n_{\text{el}}}, \boldsymbol{R}_1, \dots \boldsymbol{R}_{N_{\text{atoms}}}) = h(\boldsymbol{r}_1, \dots, \boldsymbol{r}_{n_{\text{el}}}, \boldsymbol{R}_1, \boldsymbol{R}_{N_{\text{atoms}}}). \tag{4}$$

To demonstrate the problem consider a simplified 1D example with a single electron and a supercell consisting of N primitive cells, with lattice consant a, each containing a single nucleus. The coordinates R_J of all nuclei in the supercell are thus given by

$$R_J = Ja. \tag{5}$$

An embedding satisfying the invariances eq. (2), eq. (3), eq. (4) is given by any permutation invariant function h

$$h = h\left((\omega(r - R_1), \dots, \omega(r - R_{N_{\text{atoms}}})\right) \tag{6}$$
$$= h(\omega(r - a), \omega(r - 2a), \dots, \omega(r - Na)), \tag{7}$$

where ω computes input features that are periodic in the supercell

$$\omega(x) = \omega(x + L_{\text{sc}}) = \omega(x + Na). \tag{8}$$

Here using distances $r - R$ automatically enforces eq. (2) and using periodic versions $\omega(r - R)$ of these distances automatically enforces eq. (4).

For this system, any embedding following the structure in eq. (6) is necessarily not only invariant under translations of electrons by a *supercell* lattice vector, but also invariant under translation of

electrons by a *primitive* lattice vector.

$$
\begin{aligned}
h_{\text{prim}}^{\text{shifted}} &= h(r+a, R_1, \dots, R_{N_{\text{atoms}}}) && (9)\\
&= h\left((\omega(r+a-a), \omega(r+a-2a), \dots, \omega(r+a-Na)\right) && (10)\\
&\overset{3}{=} h\left((\omega(r-a), \omega(r-2a), \dots, \omega(r)\right) && (11)\\
&\overset{4}{=} h\left((\omega(r-a), \omega(r-2a), \dots, \omega(r-Na)\right) && (12)\\
&= h(r, R_1, \dots, R_{N_{\text{atoms}}}) = h^{\text{orig}}. && (13)
\end{aligned}
$$

Therefore using electron embeddings with these symmetries allows only representation of orbitals that are periodic on the primitive lattice. This excludes many relevant functions such as localized orbitals and prevents the network from representing long-range correlations. To break this unwanted symmetry there are at least three options:

- Break invariance with respect to permutation of nuclei. Non-transferable ansatze such as FermiNet [6], PsiFormer [8] or DeepSolid [5] all break this permutation invariance. Since these approaches are only ever trained on a single system (and thus a single permutation of nuclei) this poses no issue there, but prevents efficient generalization to permuted, but physically identical systems.
- Break supercell lattice translational symmetry. For gas-phase calculations there is no periodicity and thus existing transferable approaches [9–11] do not face this issue. For periodic systems however periodicity is required to be able to enforce boundary conditions.
- Use permutation *equivariant* electron-ion embeddings instead of permutation *invariant* electron embeddings, as done in this work.

5 Basis set dependence

We do not observe a dependence of the energies obtained by our method on the basis set used to generate the orbital features, as long as the basis is sufficient to allow a qualitatively correct model of the mean field orbitals. Fig. 5 demonstrates this on the example of graphene in a 1×1 supercell. While the Hartree-Fock energy strongly depends on the basis set size, our energy after variational optimization is basis-set independent. Even in a minimal basis set like STO-6G, our method yield identical energies.

6 Hyperparameters

A detailed description of the hyperparameter used in this work can be found below (cf. supplementary Table 2). For optimization we rely on the second-order method KFAC [12] and use their Python implementation [13].

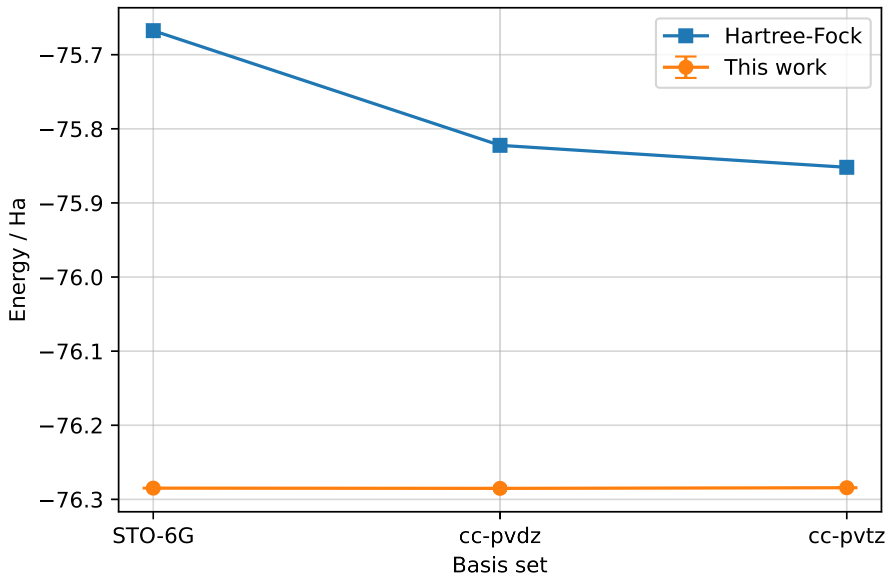

Supplementary Figure 5: **Basis set dependence**: Twist-averaged energy for graphene in a 1×1 supercell as a function of basis set size. While the Hartree-Fock energy strongly depends on the basis set, the variational energies obtained from our method are basis-set independent.

7 Total energies

For better comparison we include the individual total energies for the results in Tab. 1 and Fig. 5. For graphene we state the total energies per primitive cell for each twist plus the combination of structure-factor-based finite size corrections and ZPVE in Supplementary Table 3. For LiH we state the twist averaged energies per primitive cell for each lattice constant, separately depicting structure-factor-based finite size correction and ZPVE in Supplementary Table 4. For LiH the zero-point vibrational energies (ZPVE) are taken from [14].

8 Variance of energies

Fig. 6 depicts the variance of the local energies for two systems, the Γ-point calculations for varying lattice constances of LiH in a $2 \times 2 \times 2$ supercell as well as for three twists of Graphene in a 2×2 supercell for DeepSolid and our work. In both cases the variance converges towards zero over training epochs.

We smooth the optimization curves by applying a running average over 1000 steps for LiH and 4000 steps for graphene. We note that Monte Carlo outliers can cause spikes in the energy, as is for example visible in Supplementary Figure 6 at ≈80k steps. The DeepSolid data was taken from the github repository by Li [15]. One of the optimization curves of the $2 \times 2 \times 2$ supercell of LiH misses ≈ 380 optimization steps out of the 300k steps. These missing steps were excluded from the running average.

9 Reuse from Independent Calculations

Fig. 7 compares a shared calculation across lattice constances and twists for a $2 \times 2 \times 2$ LiH supercell with fine-tuned calculations from a pre-trained single calculation run. The fine-tuned calculation uses a pre-trained model from the Γ-point of the equilibrium geometry with 100k pre-training steps. It can be seen that the shared optimization approach converges faster and slightly lower compared to reusing from independent.

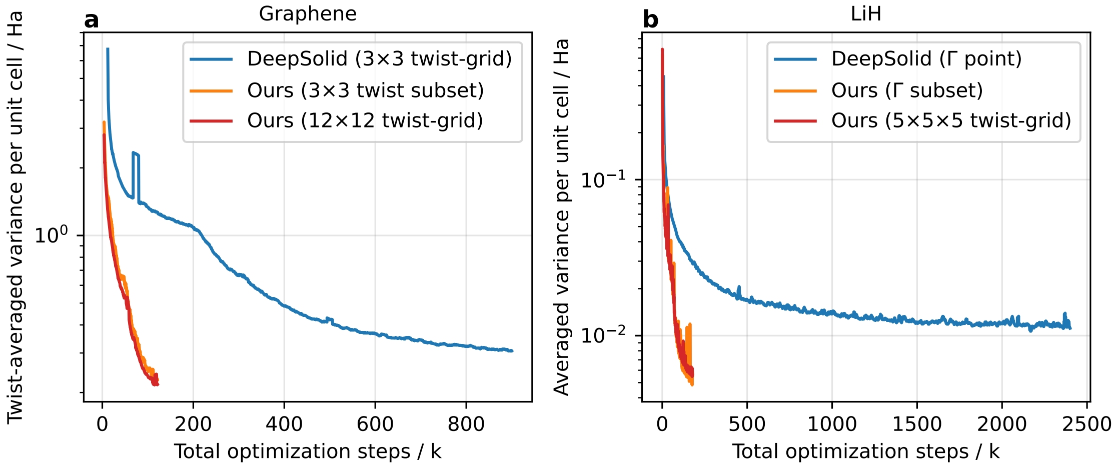

Supplementary Figure 6: **Variance of energies**: Variance of the local energies for the Γ-point calculations of LiH in a $2 \times 2 \times 2$ supercell with varying lattice constances and three twists of the Graphene 2×2 supercell plotted over number of epochs for DeepSolid and our work.

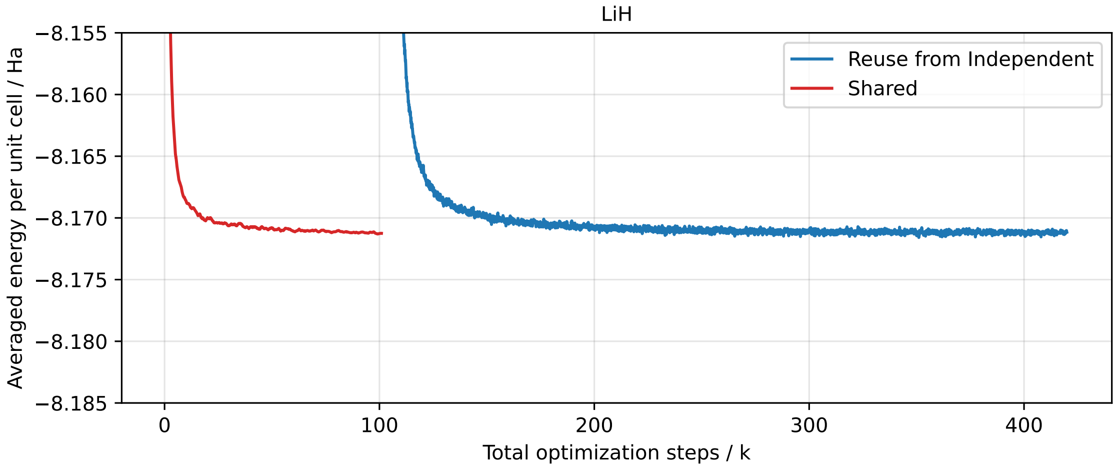

Supplementary Figure 7: **Reuse from an independent calculation for LiH**: The aggregated twist averaged energy over all geometries is displayed for a shared calculation and independetly fine-tuned calculations that use a pre-trained model Γ-point of the equilibrium geometry with 100k pre-training steps.

Supplementary Table 2: Hyperparameter settings used in this work

**HF-pre-training**	Pre-training basis set	cc-pVDZ
	Pre-training steps per geometry	100-500
**Embedding**	Envelope power γ	2
	Uniform initialization of envelope scaling α	8-10
	El-el hidden dimension	32
	El-Ion hidden dimension	128
	Ion hidden dimension	128
	№ hidden layers of ion embedding	3
	Final el-ion embedding dimension e_{iI}	64
**Transferable atomic orbitals**	№ determinants n_{det}	8
	Basis set for orbital descriptor	cc-pVDZ
	№ hidden layers of f^W	2
	Hidden dimension of f^W	128
	№ hidden layers of f^a	2
	Hidden dimension of f^a	32
	Activation function	ReLU
	Residual connection	True
	Layer Norm	True
**Jastrow factor**	№ hidden layers of MLP	2
	Hidden dimension of MLP	40
**Markov Chain Monte Carlo**	№ walkers	2048
	№ decorrelation steps	20
	Target acceptance prob.	50%
**Variational optimization**	Optimizer	KFAC
	Damping	$1 - 3 \times 10^{-3}$
	Norm constraint	1×10^{-3}
	Batch size	2048
	Initial learning rate lr_0	$0.1 - 0.3$
	Learning rate decay	$\text{lr}(t) = \text{lr}_0(1 + t/6000)^{-1}$
	Optimization steps	100,000 - 200,000
**Changes for Reuse**	Initial learning rate lr_0	0.05
	Learning rate decay	$\text{lr}(t) = \text{lr}_0(1 + t/6000)^{-1}$
	Optimization steps	0 - 10,000

10 Seed Dependence of the Calculations

As shown in the work by Gerard et al. [16] we do not expect a large deviation between runs and therefore seed each calculation randomly.

Supplementary Table 3: **Total energies of graphene** in Hartrees. The energies depict the total energies per primitive cell by sequentially adding structure-factor-based finite-size correction (SFC) and ZPVE. The systems represent the symmetry-inequivalent twists for the 12 × 12 Monkhorst-Pack grid.

**Twists**	**Total Energy**	**Total energy + SFC**	**Total energy + SFC + ZPVE**
$k = (0.33, 0.33)$	−76.2572	−76.2543	−76.2415
$k = (0, -0.17)$	−76.2002	−76.1972	−76.1844
$k = (-0.08, -0.42)$	−76.2679	−76.2650	−76.2522
$k = (-0.08, -0.33)$	−76.2470	−76.2441	−76.2313
$k = (-0.08, -0.25)$	−76.2190	−76.2160	−76.2032
$k = (-0.17, -0.42)$	−76.2615	−76.2586	−76.2458
$k = (0.67, 0.33)$	−76.2590	−76.2560	−76.2432
$k = (-0.17, -0.58)$	−76.2775	−76.2746	−76.2618
$k = (-0.08, -0.17)$	−76.1914	−76.1884	−76.1756
$k = (-0.08, -0.50)$	−76.2786	−76.2757	−76.2629
$k = (0, 0)$	−76.1572	−76.1542	−76.1414
$k = (0, -0.25)$	−76.2308	−76.2278	−76.2150
$k = (-0.17, -0.33)$	−76.2407	−76.2377	−76.2249
$k = (-0.25, -0.50)$	−76.2688	−76.2658	−76.2530
$k = (0, -0.08)$	−76.1723	−76.1693	−76.1565
$k = (0, -0.50)$	−76.2787	−76.2758	−76.2630
$k = (0, -0.42)$	−76.2737	−76.2708	−76.2580
$k = (-0.25, -0.58)$	−76.2728	−76.2699	−76.2571
$k = (-0.17, -0.50)$	−76.2740	−76.2710	−76.2582
Averaged 3 × 3	−76.2465	−76.2435	−76.2307
Averaged 12 × 12	−76.2503	−76.2473	−76.2345

Supplementary Table 4: **Total energies of LiH** in Hartrees. The energies depict the twist averaged total energies per primitive cell by sequentially adding structure-factor-based finite-size correction (SFC) and ZPVE. For the twist averaging we use a 5 × 5 × 5 Monkhorst-Pack grid per lattice constant. The energies accompany the Figure 5.

**Supercell size**	**Lattice constant / a_0**	**Total Energy**	**Total energy + SFC**	**Total energy + SFC + ZPVE**
2 × 2 × 2	6.4	−8.1654	−8.1462	−8.1338
	6.8	−8.1752	−8.1574	−8.1464
	7.2	−8.1792	−8.1626	−8.1529
	7.6	−8.1789	−8.1636	−8.1554
	7.9	−8.1759	−8.1618	−8.1545
	8.3	−8.1709	−8.1580	−8.1522
	8.7	−8.1646	−8.1527	−8.1482
	9.1	−8.1574	−8.1467	−8.1436
3 × 3 × 3	7.674	−8.1644	−8.1604	−8.1524

References

[1] Simons Collaboration on the Many-Electron Problem et al. "Towards the Solution of the Many-Electron Problem in Real Materials: Equation of State of the Hydrogen Chain with State-of-the-Art Many-Body Methods". In: *Physical Review X* 7.3 (Sept. 2017), p. 031059. DOI: 10.1103/PhysRevX.7.031059.

[2] Simons Collaboration on the Many-Electron Problem et al. "Ground-State Properties of the Hydrogen Chain: Dimerization, Insulator-to-Metal Transition, and Magnetic Phases". In: *Physical Review X* 10.3 (Sept. 2020), p. 031058. DOI: 10.1103/PhysRevX.10.031058.

[3] Leo Brewer. *LBL3720: The Cohesive Energies of the Elements.* Tech. rep. Lawrence Berkeley Laboratory, 1977.

[4] S. Lebègue et al. "Cohesive Properties and Asymptotics of the Dispersion Interaction in Graphite by the Random Phase Approximation". In: *Physical Review Letters* 105.19 (Nov. 2010), p. 196401. DOI: 10.1103/PhysRevLett.105.196401.

[5] Xiang Li, Zhe Li, and Ji Chen. "Ab Initio Calculation of Real Solids via Neural Network Ansatz". In: *Nature Communications* 13.1 (2022), p. 7895. DOI: 10.1038/s41467-022-35627-1.

[6] David Pfau et al. "Ab Initio Solution of the Many-Electron Schrödinger Equation with Deep Neural Networks". In: *Phys. Rev. Res.* 2.3 (2020), p. 033429. DOI: 10.1103/PhysRevResearch.2.033429.

[7] Simone Chiesa et al. "Finite-Size Error in Many-Body Simulations with Long-Range Interactions". In: *Physical Review Letters* 97.7 (Aug. 2006), p. 076404. DOI: 10.1103/PhysRevLett.97.076404.

[8] Ingrid von Glehn, James S Spencer, and David Pfau. "A Self-Attention Ansatz for Ab-Initio Quantum Chemistry". In: *The Eleventh International Conference on Learning Representations.* 2023.

[9] Nicholas Gao and Stephan Günnemann. "Generalizing Neural Wave Functions". In: *Proceedings of the 40th International Conference on Machine Learning.* Vol. 202. Proceedings of Machine Learning Research. PMLR, 2023, pp. 10708–10726.

[10] Michael Scherbela, Leon Gerard, and Philipp Grohs. "Towards a Transferable Fermionic Neural Wavefunction for Molecules". In: *Nature Communications* 15.1 (2024), p. 120. DOI: 10.1038/s41467-023-44216-9.

[11] Michael Scherbela, Leon Gerard, and Philipp Grohs. "Variational Monte Carlo on a Budget - Fine-Tuning Pre-Trained Neural Wavefunctions". In: *Thirty-Seventh Conference on Neural Information Processing Systems.* 2023.

[12] James Martens and Roger Grosse. "Optimizing Neural Networks with Kronecker-factored Approximate Curvature". In: *Proceedings of the 32nd International Conference on Machine Learning.* PMLR, June 2015, pp. 2408–2417.

[13] Aleksandar Botev and James Martens. *KFAC-JAX.* 2022. URL: https://github.com/google-deepmind/kfac-jax.

[14] S. J. Nolan et al. "Calculation of Properties of Crystalline Lithium Hydride Using Correlated Wave Function Theory". In: *Phys. Rev. B* 80.16 (Oct. 2009), p. 165109. DOI: 10.1103/PhysRevB.80.165109.

[15] Xiang Li. *DeepSolid simulation data.* https://github.com/GiantElephant123/solid_simulation_data. Accessed: 2025-07-18. 2025.

[16] Leon Gerard et al. "Gold-Standard Solutions to the Schrödinger Equation Using Deep Learning: How Much Physics Do We Need?" In: *Advances in Neural Information Processing Systems.* Vol. 35. Curran Associates, Inc., 2022, pp. 10282–10294.
